# Barriers and facilitators to developing faith-based peer interventions in Islamic religious settings for obesity prevention in women: A qualitative exploratory study

**DOI:** 10.1371/journal.pone.0340087

**Published:** 2026-01-05

**Authors:** Maha Shafique, Salman Waqar, Sundus Mahdi

**Affiliations:** 1 Department of Public Health, Environments, and Society, London School of Hygiene and Tropical Medicine, London, United Kingdom; 2 Department of Primary Care and Public Health, Imperial College London, London, United Kingdom; 3 Department of Health Sciences, University of York, York, United Kingdom; Emory University, School of Public Health, UNITED STATES OF AMERICA

## Abstract

**Background:**

Muslims represent the second-largest religious group in England and are geographically concentrated in socioeconomically deprived areas, predisposing them to health disparities. Faith-based interventions in Muslim communities are under-researched, despite their potential to promote health among hard-to-reach groups. This study investigated the barriers and facilitators to developing faith-based peer interventions aimed at reducing obesity-related health inequalities among South Asian Muslim women.

**Methods:**

Using a qualitative approach, the study involved semi-structured interviews with nine Muslim women and six religious leaders of South Asian heritage, in Walsall, England. Interviews explored women’s health beliefs, the community milieu, attitudes towards faith-based health interventions and experiences of health-related activities in religious settings. The data was analysed thematically, which guided the development of a logic model for potential faith-based interventions.

**Results:**

Socio-cultural factors may shape women’s health behaviours, gender constructs, the organisational dynamics of religious settings, and women’s involvement therein. All participants supported the integration of faith-based elements into future health interventions. While most participants favoured peer-led approaches, some recommended holistic health education by health professionals and religious leaders. Proposed peer strategies for prospective interventions included support and social groups, educational workshops, counselling, and group physical activities. Effective interventions require dynamic leadership, organisational partnerships, stable funding, and volunteer training. Friendliness, trustworthiness, competency, and positive role modelling were highlighted as desirable peer characteristics.

**Conclusion:**

Faith-based peer interventions could serve as a culturally and religiously sensitive approach to address obesity-related inequalities in this demographic. Peer-based approaches enhance community engagement, social networks, and self-efficacy, potentially influencing socio-cultural norms. The success of future interventions would depend on co-production, reorganising religious spaces as community hubs, fostering inclusivity, and encouraging female leadership.

## 1. Introduction

Muslims are the second-largest religious group in England constituting 4.8% of the population, largely of South Asian (SA) descent [1]. The English Indices of Deprivation revealed that 46% of Muslims reside in the most deprived 10% of neighbourhoods across England [2]. Deprivation is associated with obesity and reduced life expectancy [3]. Obesity, a major modifiable risk factor for noncommunicable diseases [4], is particularly prevalent among SAs in the UK [5,6], and is thought to be multifactorial from differences in body composition and metabolism [5,7,8], dietary practices [9,10], and socioeconomic disparities [9,11]. SA populations have a high predisposition to visceral obesity [6,7] and can develop insulin resistance at a lower body mass index (BMI) compared to other ethnic groups [7,8]. They tend to consume diets rich in sugar, saturated fats, and salt, and consume fewer fruits and vegetables [10,12], leading to a net positive energy balance. Obesity-related health inequalities are more pronounced in women, with a 20% difference in obesity prevalence between England’s most and least deprived areas for women, compared to 8% for men [11]. Furthermore, Muslim women may have lower levels of outdoor physical activity due to sociocultural and religious factors, such as modesty norms [13]. For instance, a recent national survey indicated that Muslims and Asian women exhibit the highest rates of physical inactivity within the population [14].

The COVID-19 pandemic exacerbated existing health disparities leading to poorer health outcomes and a high psychosocial burden [15,16], highlighting the need for tailored and targeted community-centred approaches to obesity prevention [17,18]. These approaches leverage distinctive community assets to empower marginalised groups by promoting self-efficacy, social cohesion, and community ownership [17]. In Muslim communities, faith is central to community identity, with Islamic religious settings (IRS) at the heart of neighbourhoods [19]. Faith-based interventions (FBIs), which incorporate theologically tailored health messaging to promote wellbeing [18], can effectively engage these communities by tapping into their belief systems, community spirit, and religious leadership [20–22]. Small-scale studies in other religious settings have demonstrated the effectiveness of FBIs in preventing obesity, reporting positive outcomes like weight reduction [23,24], improved diets [24], and increased physical activity [23–25]. However, research in Western IRS remains limited, employing various modalities, such as sermons [26], health fairs [27], peer interventions [21,28,29], and group education [29,30]. A recent scoping review on obesity-related FBIs in IRS revealed that despite their wide reach, these are often poorly described and rarely evaluated [20].

This qualitative exploratory study examines the acceptability of developing faith-based peer interventions for obesity prevention in South Asian Muslim women (SAMW), given the high predisposition to obesity among SAs [5,6] and the dearth of research on Muslim women [20]. Muslim women may be less responsive to mainstream health interventions due to language barriers, lack of cultural competency, perceived discrimination, and concerns about gender concordance, which can delay health-seeking [31–33]. Peer interventions, where individuals with shared characteristics act in a non-professional capacity to support health initiatives [34], have shown promise in engaging minority communities, promoting lifestyle education [35], smoking cessation [36], noncommunicable disease management [37], and mental health awareness [38,39]. Research suggests that Muslim women may respond positively to faith-based peer interventions [21], which have also proven effective in improving cancer screening uptake [28,29].

Recognising the potential of FBIs to reach underserved groups [40,41], this study explored approaches to obesity intervention in this demographic. Specifically, the study (1) assessed the beliefs and lifestyle behaviours of SAMW in relation to obesity and examined the influence of socioeconomic, cultural, and religious factors on their behaviours, (2) investigated the perspectives of SAMW and religious leaders (RLs) on the acceptability, barriers, and facilitators to developing faith-based peer interventions in IRS, and (3) made strategic recommendations for developing FBIs using a logic model.

## 2. Methods

### 2.1. Design

This qualitative cross-sectional study employed semi-structured, one-to-one interviews to facilitate a nuanced analysis of the participants’ worldviews and the social context. It was set in Walsall, home to a large Muslim population of 11.3% [42], and one of the most disadvantaged areas in England with a growing socioeconomic divide [2]. It was approved by London School of Hygiene and Tropical Medicine Research Ethics Committee (Ref: 28509).

### 2.2. Recruitment

Between 1^st^ July and 13^th^ September 2023, two participant groups (RLs and SAMW) were recruited using purposive sampling to capture sample diversity, response variability, and include information-rich participants (RLs) [43]. All participants were of SA origin – specifically Pakistani, Indian, and Bangladeshi – the three largest Muslim ethnic groups in England [1].

RLs were defined as individuals holding leadership, management, or educational roles in IRS, and were considered key informants due to their knowledge and influence [19]. The inclusion criteria for RLs were (a) male or female, (b) over 18 years old, (c) affiliated with a Walsall-based IRS, and (d) having basic English literacy. Six RLs were recruited via invitation letters from diverse IRS (mosques, Muslim community centres, seminary) based on their key role in the setting, with equal male and female representation to ensure a balanced sample. Due to cultural norms limiting direct access to male RLs and to secure a higher response rate, a trusted male contact distributed invitation letters in person, while female RLs were approached directly by the primary researcher (MS). All RLs who were contacted agreed to participate in the study.

The inclusion criteria for women were (a) South Asian (b) Muslim (c) over 18 years old, (d) Walsall resident, and (e) having basic English literacy. Nine SA women of different age groups were recruited purposively using invitation flyers, social media platforms, social connections, and snowball sampling. As flyer responses were low, snowball sampling and social connections were most effective for recruiting women of different backgrounds. The sample size was small due to pragmatic reasons as an explorative study; and the recruitment period was time-limited as the study was conducted as part of a postgraduate dissertation.

### 2.3. Participants and data collection

The twelve female respondents (nine SAMW and three female RLs) completed a sociodemographic questionnaire before the interviews (*see* Table 1). While education, marital and employment status were not part of the sampling criteria, this information helped contextualise the interview data on women’s health beliefs and behaviours. The questionnaire also confirmed that participants met the inclusion criteria and facilitated the recruitment of a diverse participant group. As the study focused exclusively on women’s health behaviours, male RLs were not asked to complete this questionnaire.

**Table 1:** Female participants’ characteristics (n = 12).

Characteristic	Categories	n (%)
Age (years)	18-29	2 (17%)
	30-39	4 (33%)
	40-49	4 (33%)
	50-59	1 (8%)
	60-65	1 (8%)
Marital status	Single	2 (17%)
	Married (without children)	2 (17%)
	Married (with children)	6 (50%)
	Divorced	1 (8%)
	Widowed	1 (8%)
Education	Less than high school degree	1 (8%)
	High school degree or equivalent	3 (25%)
	Bachelor’s degree	4 (33%)
	Master’s degree	1 (8%)
	Other (Islamic studies)	3 (25%)
Employment status	Student	0
	Not employed	2 (17%)
	Employed, working part-time.	5 (41%)
	Employed, working full-time.	2 (17%)
	Other (e.g., freelancer, self-employed)	3 (25%)
Ethnic groups	Indian	7 (58%)
	Pakistani	4 (33%)
	Bangladeshi	1 (8%)

All data are presented as n (% of the whole group of participants).

The interview guides consisted of 15–20 open-ended questions, tailored for SAMW, male RLs and female RLs (*see supplementary* S1 File*)*. They included illustrative questions and probes; however, the order and wording of questions was flexible and guided by the discussion. The interview guide for SAMW was pilot tested with a local Muslim woman to assess its appropriateness and acceptability; based on this, some questions were reworded for clarity and neutrality.

To establish rapport, interviews began with warm-up questions about participants’ backgrounds, roles and responsibilities. SAMW were then asked about their health beliefs, lifestyle, social determinants, satisfaction with health-related activities in IRS, and thoughts on FBIs and peer-led activities. Male RLs discussed the social context, the role of faith and IRS, the acceptability and feasibility of FBIs for women, views on peer interventions, and local partnerships. Female RLs were interviewed about all the aforementioned themes. At the end of each interview, participants were invited to share additional comments or feedback.

The researcher’s social identity and positionality in relation to the interviewees influence rapport and the generated data [43]. Interviews were conducted by MS, a local South Asian Muslim female of Pakistani origin, fluent in English and Urdu. Participants were given a detailed information sheet before the interviews to explain the study and the researcher’s background, following which informed written consent was obtained from all participants. Of the fifteen interviews, five were held in-person at community-based settings, while ten were arranged virtually (Zoom), based on participants’ preferences. Appropriate timings were chosen to maintain privacy. Interviews lasted between 30–90 minutes, were conducted in English, and audio recorded.

Interview recordings were transcribed verbatim (by MS), including any emotional expressions, fillers, and non-lexical sounds, but omitting repetitive words to facilitate smoother reading. Any Urdu or Arabic phrases were translated into English (by MS). Transcripts were de-identified using pseudonyms and each transcript was assigned a unique numeric identifier. Participant characteristics and interview details (date, duration) were recorded separately. All transcripts were cross-checked against recordings to verify accuracy. After each interview, field notes were recorded to document reflections and ideas. Data analysis was carried out iteratively alongside data collection to allow for refinement of the topic guide and recruitment of further participants to explore emerging patterns, such as the sociocultural barriers to organising faith-based interventions across different settings.

### 2.4. Analysis

Thematic analysis [43] was conducted by MS using *Atlas.ti* software to identify patterns (themes) within data and understand concepts from the participants’ viewpoints [43]. The process began with data familiarisation by reviewing interview transcripts, developing interview summaries, and recording initial data patterns. Codes were then assigned to data segments that represented important or recurring themes. A provisional codebook was developed, categorising codes broadly around the research objectives. Following a mixed inductive-deductive approach [43], the codes concerning SAMW’s beliefs and behaviours were organised under four themes – individual, interpersonal, community and societal – based on the socio-ecological framework of health, which accounts for cultural, religious, and socioeconomic influences on health behaviours [44]. The codes relating to the acceptability, barriers, facilitators, and strategies for FBIs were developed inductively, although some overarching themes were informed by the interview guide. For instance, the theme “peer characteristics” was identified from the interview guide, while the codes within it (e.g., role modelling, training) emerged inductively.

The provisional codebook was initially applied to a few transcripts to see how well the codes captured the data, and was continuously revised by merging existing codes and generating additional codes to create a final code structure. All transcripts were coded using the final codebook to ensure consistency. Data inconsistencies and unusual viewpoints were noted for a transparent analysis. A coding tree summarising the major themes and codes was developed *(see supplementary* S1 Fig), complemented with illustrative interview excerpts *(see* *supplementary* S1 Table*)*. Findings from the thematic analysis were subsequently synthesised to inform a logic model [45] for developing FBIs in IRS. Reporting followed COREQ guidelines *(see supplementary* S2 File*)* [46]*.*

## 3. Results

### 3.1. Factors influencing the health behaviours of SAMW

**Fig 1:**
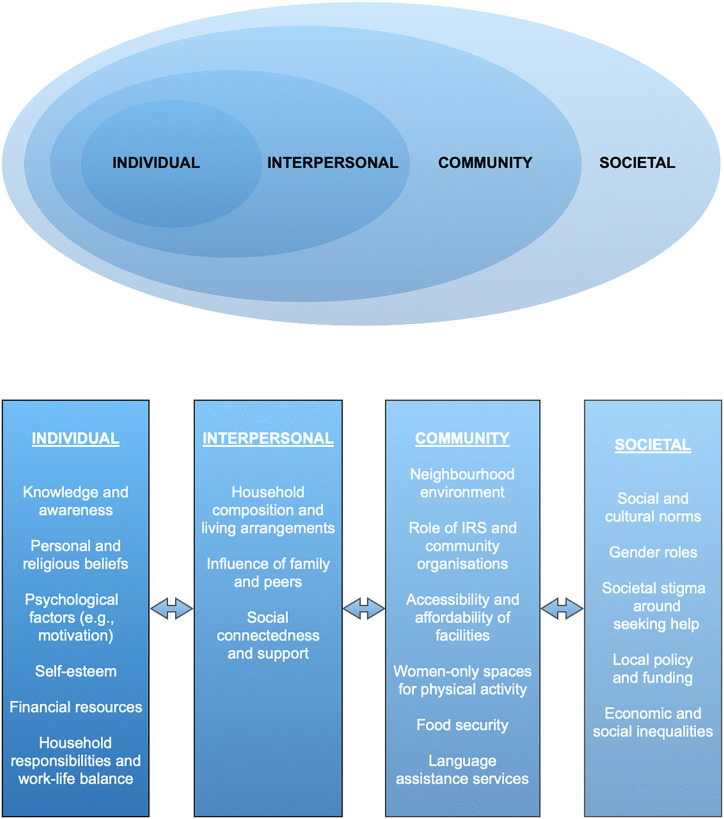
The socioecological model to conceptualise the determinants of health behaviours of South Asian Muslim women at four levels. The boxes encompass themes that emerge from the interview data. The bidirectional arrows represent the dynamic interactions between the individual and the broader environment. *Adapted from CDC (2007)* [47].

The key determinants of SAMW’s health behaviours were mapped using the socioecological model (*see* Fig 1).

#### 3.1.1. Individual and interpersonal factors.

Faith was seen as a positive influence on health behaviours, with respondents affirming that Islamic teachings promote balance, self-care, and healthy practices like intermittent fasting, portion control, physical activity, and personal hygiene. All female interviewees viewed their bodies as a trust from God, and Prophet Muhammad as their role model.

*“I’m motivated by my religion to eat healthy…Prophet [Muhammad]…would encourage exercises…sports, horse riding, archery… [Our religion] talks about giving yourself time…”* ***(Participant 2, 40-49, Teacher)***

However, these teachings may not always filter into people’s lives, as they may selectively follow them or remain incognizant*.* One RL noted that some community members hold a fatalistic attitude, neglecting preventative health care while believing that illness is predetermined by God.

*“What we [the community] are not good at is working on prevention, and I think it’s partly to do with our belief that regardless of what we do, it is going to happen to us [destiny].”* ***(Participant 13, Male RL)***

SAMW reported a lack of health education by medical providers, language barriers, and cultural dietary practices as barriers to healthy eating.

*“Culture is the key factor. You’re used to something already, so you don’t question it. You’re used to a certain lifestyle, and you find nothing wrong with it.”* ***(Participant 1, 38–39, Homemaker)***

They also discussed feelings of peer pressure, people pleasing, and difficulty resisting unhealthy foods, especially in joint households and social gatherings.

*“If I was in my own space, I’d be more disciplined because I’ve got less distractions and influences around me, less people pleasing…”* ***(Participant 3, 30-39, Teacher)***

Motivation was a common challenge for women, like consistently following a meal plan, managing unhealthy cravings, exercising regularly, and resisting emotional eating during stressful periods. Additionally, balancing household chores with work or family responsibilities left many women overwhelmed, leading to neglect of self-care, especially for those with extensive caregiving duties.

Conversely, participants with supportive family or friends felt it was easier to make healthier lifestyle choices.

*“I cook for my family, so I’m in charge…It’s very easy for me to change the kind of cooking, and no one minds...”* ***(Participant 2, 40-49, Teacher)***

#### 3.1.2. Community and societal factors.

RLs noted the decline of council-led health programs and resources due to funding cuts, leaving private facilities, like gyms, unaffordable for many. Most women expressed dissatisfaction with local health and recreational facilities, citing poor maintenance and closure of community centres such as “Sure Start”, which had been offering well-being services for families with young children.

*“[Sure Start] was a vital service…You were educating mothers about the early years, which led to better habits, lifestyles, choices and routines within the family unit…”* ***(Participant 9, 18-29, Female RL)***

RLs also highlighted the government’s lack of engagement with multi-ethnic communities, noting that a blanket approach marginalises minority groups and erodes trust in the healthcare system, hindering their participation in health interventions. One RL referenced the example of COVID-19, which disproportionally affected ethnic minorities. It was proposed that these barriers to engagement could be overcome by collaborating with local communities to design tailored services.

*“…Reaching out to these communities, acknowledging their discomfort, working with them to come up with solutions… What works for you?...Whether that be sessions for religious communities separately…at their places of engagement…in their languages…”* ***(Participant 9, 18-29, Female RL)***

Women reported a lack of easily available information about local facilities and suggested creating a digital platform that provides updated information. They also raised concerns about accessibility of facilities, such as language barriers and cultural sensitivity. For instance, some women expressed the need for women-only spaces for physical activity, where they could dress modestly without fear of judgement, citing past negative experiences,

*“When I went to gym [in religious attire]…people were [staring at me] as if I am here to show some circus…I never went back again.* ***(Participant 12, 30-39, Homemaker)***

RLs also observed the lower participation rates of Muslim women in council-led outdoor activities, like walking groups, probably due to feeling insecure as a visibly covered Muslim.

*“Our women, especially if they wear hijab (head covering), feel vulnerable, they don’t feel safe....”* ***(Participant 13, Male RL)***

Neighbourhood issues, such as poorly maintained and littered streets, and safety concerns also discouraged outdoor activities. Many women felt unsafe going out after dark due to the pervasiveness of anti-social behaviour, drugs, and street crime.

*“…As a female who doesn’t drive…we don’t really live in a safe area…I’d be fearful of something happening…”* ***(Participant 3, 30-39, Teacher)***

Easy access to unhealthy foods was another concern, with cheap takeaways proliferating in ethnic minority neighbourhoods, and unhealthy snacks displayed at supermarket tills. Healthy options were considered less affordable due to the rising cost of living.

*“I know personally in the community so many people [who] struggle to buy ingredients which are good for health. Even if you go to food banks, they have tinned foods...”* ***(Participant 5, 40–49, Entrepreneur)***

One RL also noted the stigma around seeking financial help in SA communities, which compounded the struggles faced by low-income families.

### 3.2. Acceptability of faith-based peer interventions in IRS

#### 3.2.1. Integrating faith-based elements in health interventions in IRS.

All respondents supported integrating faith-based elements into health interventions. RLs observed that people often overlook the link between health and scriptural teachings and emphasised the importance of reinforcing this connection. SAMW highlighted the need for RLs to address health-related topics consistently in their speeches.

All interviewees supported utilising IRS for health promotion, viewing these as accessible, positive spaces for Muslims, and potential “one-stop” hubs for health and spirituality. Some women expressed feeling more comfortable engaging in IRS-based health initiatives compared to other community settings.

*“In a religious space…we’re allowed to feel as if we’re all the same…I would definitely…feel a lot more comfortable.”* ***(Participant 8, 20-29, Teacher)***

Respondents believed that trusted health professionals could complement faith-based teachings with scientific evidence. Many health professionals attend daily prayers, and mosques were viewed as ideal for promoting health initiatives, making it easier to advertise programs*,* network effectively, and reach a specific audience, particularly those less likely to access other venues.

*“…Majority of the people are more linked with mosques than other organisations…If you’ve got 500 people coming to mosque daily, to promote all activities…is much easier…”.* ***(Participant 14, Male RL)***

RLs shared examples of successful past events held in IRS with support from health professionals, including cancer and mental health awareness, life-saving skills, blood donation drives, and health checks. However, such programs were limited to certain IRS whose leadership prioritised health-related activities. These initiatives were infrequent due to resource, time, and logistical constraints. RLs noted that women’s attendance at health events was encouraging, and that they preferred gender-segregated arrangements.

*“We tend to run them (events) separately because we find that women are more comfortable about opening up and questioning the professionals there...”* ***(Participant 11, 30-39, Female RL)***

#### 3.2.2. Acceptability of peer-based approaches.

Majority of participants supported peer-based approaches due to shared background, language, and values with peers. Women perceived “peers” as individuals with shared culture, religion, and language, not necessarily similar social or health status. Due to commonality, respondents also viewed SA Muslim health experts as professional peers. Peer approaches were seen as helping women feel more comfortable and confident in seeking help.

*If you were to have somebody from within your own community…who has shared your experiences.... who speaks your language…I think that becomes a very natural, trusting experience…”* ***(Participant 9, 18–29, Female RL)***

A minority of SAMW believed that peer-based approaches were unnecessary if the intervention was led by a trustworthy, knowledgeable, and culturally aware health professional. However, they noted that advice from someone unfamiliar with their culture might be harder to follow. For instance, one respondent shared her experience of joining a fitness group led by a European Muslim coach, where she struggled to follow the diet plans.

*”I found it very challenging because I’m used to our Asian foods…I didn’t enjoy the taste…”* ***(Participant 6, 50-59, Receptionist)***

### 3.3. Barriers and facilitators to faith-based peer interventions in IRS

#### 3.3.1. Leadership.

RLs underscored the importance of visionary leadership, and the influence of cultural factors on the organisational structure and priorities of IRS. Some RLs, holding traditional views, may view mosques solely as places of worship and religious education, deprioritising health projects. RLs advocated for mosques to transform into multipurpose community hubs, aligning with their historical role in the Islamic tradition.

*“In the time of Prophet Muhammad …(mosque) wasn’t just a place of prayer and contemplation…it was the hub of the community.”* ***(Participant 14, Male RL)***

RLs also noted that IRS are volunteer-led and self-financed, with limited resources to handle various community issues. Health projects may be sidelined due to resource constraints, frequent team changes, and volunteer time limitations.

*“Our mosques are already so burdened by the number of (community) issues they have to deal with day in and day out that health goes very far down the list.”* ***(Participant 11, 30-39, Female RL)***

To address this, RLs suggested forming a dedicated “health and wellbeing” team within the IRS management committee. They also noted a lack of cultural emphasis on disease prevention, particularly among lower-educated individuals, and a low public demand for health programmes, stressing the need to raise community health awareness.

*“Those who are in the management and leadership roles…[They] are reluctant to start these projects because they don’t see much of a demand.”* ***(Participant 14, Male RL)***

SAMW drew particular attention to the importance of modelling healthy behaviours, criticising the lack of fitness among some RLs, and the unhealthy foods typically served at IRS events.

*“…Most of the Imams…they’re obese…They are definitely not leading by example…I’d like to see them not serve pakoras and samosas at every event.”* ***(Participant 4, 40-49, Administrator)***

#### 3.3.2. Women’s inclusion and facilities.

Female RLs observed that women have traditionally not held leadership roles in IRS, as male RLs may not encourage their involvement, and older women may feel it’s not their societal role.

*“…I remember growing up, the resources and the inclusion of women was very poor…In SA communities, there’s often a cultural mindset that men are…the ones at leadership [roles]…”* ***(Participant 9, 18-29, Female RL)***

A male RL highlighted recent efforts to make local IRS more inclusive by establishing women’s management subcommittees to oversee activities for women and youth. RLs acknowledged the dynamic contributions of women throughout Islamic history and observed that younger women are increasingly stepping into leadership roles. An interviewee highlighted the contrast between cultural gender constructs and women’s role in Islamic history.

*”[In Islamic history], women were really active in the community. Culture is… they make it [seem] that women might need to stay at home, or they can’t do anything. But…Islam is not like that.”* ***(Participant 5, 40-49, Entrepreneur)***

SAMW expressed that while IRS are convenient and favourable settings for health programmes, many lack designated women’s facilities. Although activities for women have increased over time, most women remain dissatisfied with the current facilities and support systems in IRS.

*“…It’s such a loss for the community if women are not given equal access to the things that they could be benefiting from by being an active part of the mosque… Where are they supposed to go?”* ***(Participant 11, 30-39, Female RL)***

Some women appreciated their local IRS for organising regular social activities and health awareness events. RLs described a similar variability between different IRS, attributing it to differences in cultural mindsets and leadership styles.

*“It all depends on…[the] people who lead…The imams that have a vision, they’re very active. So those [IRS] will probably have a lot of programmes…”* ***(Participant 14, Male RL)***

All RLs expressed a willingness to improve women’s facilities. One RL from a mosque that lacked women’s prayer facilities drew attention to a generational divide within their management committee, with older, more conservative leaders impeding progress in establishing women’s facilities.

*“We have challenge from the elder generation who have a different view…about what the mosque should be…They are now coming around to our way of thinking...”* ***(Participant 15, Male RL)***

SAMW also called for more flexibility in scheduling activities to accommodate women from diverse backgrounds, noting that current activities for women are typically held on weekday mornings, making it difficult for professional women to attend.

*”I’d like to see more variety…to cater to different types of women. The women that work, the women who have small children, creche facilities...”* ***(Participant 1, 38-39, Homemaker)***

#### 3.3.3. Funding and human resources.

RLs praised the community’s strong volunteering spirit but highlighted the lack of resources to recruit and train volunteers in peer roles. SAMW emphasised the need for an organised network of volunteers to utilise the community’s untapped potential.

*“We don’t have any network so that we could better our own communities… It’s not that there’s a lack of talent within the community.”* ***(Participant 1, 30-39, Homemaker)***

RLs noted that some health professionals offer free services in IRS, such as counselling. However, finding qualified individuals with an Islamic ethos is challenging, and volunteers have limited availability. To enhance volunteer motivation, RLs suggested offering incentives, like payment and skills-building opportunities. This sentiment was echoed by a SAMW:

*“If I’m [volunteering]…my family will say, ‘Where are you spending your time?’…At the end of the day, if I bring some money, they won’t say anything to me.”* ***[Participant 12, 30-39, Homemaker]***

RLs noted that IRS largely rely on generous community donations, driven by the strong spiritual connection people have to these spaces. However, they emphasised the need for additional funding to regularly organise health programs. IRS-based events are typically free, and limited financial resources can restrict the capacity to host such events and provide wellbeing facilities, like exercise equipment.

*“Running promotional and health events is not cheap…You need…space, appropriate resources… We are not getting (government) funding.”* ***(Participant 9, 18-29, Female RL)***

Some RLs admitted to lacking knowledge about available funding sources, with certain grants, like lottery funding, conflicting with religious values. It was suggested that charging a small fee from participants could help sustain health programs. One RL proposed collaborating with credible, strategic Muslim organisations and charities to obtain religiously compliant, sustainable funding. Additionally, it was noted that funding bodies often favour organisations that promote inclusivity, which can pose challenges for IRS that lack adequate women’s facilities.

*“…Those mosques that are not open to the to the whole community, they find it difficult to access public funds.”* ***(Participant 13, Male RL)***

#### 3.3.4. Building community networks.

RLs advocated for increased collaboration with other IRS, local organisations, public health bodies, and the council to develop a shared vision for addressing health issues. They acknowledged the crucial role of council support and funding during the COVID-19 pandemic, which enabled the training of community champions to promote vaccine uptake through IRS. However, RLs expressed concerns that the council often neglects their community’s health needs and highlighted the lack of local representation. They proposed that Muslim umbrella organisations could help bridge this gap, and that individuals with natural leadership skills could be trained to advocate effectively for the community.

*” We need active organisations in the SA communities to work with public health and put pressure on the service providers…to be active in this area, much more than they currently are.”* ***(Participant 13, Male RL)***

One female RL highlighted the importance of cultural competency in partnerships, sharing her positive experience of collaborating with a “Sure Start Children’s Centre” on health initiatives for women and children.

*“We were very comfortable with them even though we wore [face veils] …They were very friendly, accommodating…A non-judgemental space...”* ***(Participant 10, 40-49, Female RL)***

### 3.4. Strategies for faith-based interventions

**Fig 2:**
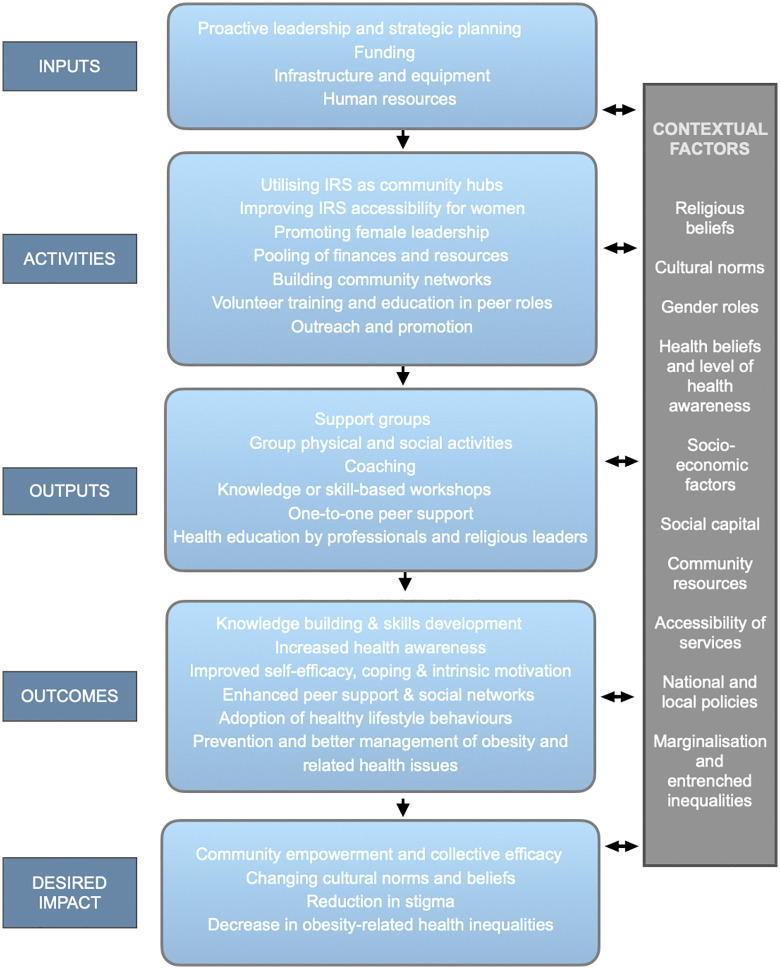
Proposed faith-based peer intervention strategies and content. Peer and intervention characteristics can influence the acceptability and effectiveness of interventions.

Participants proposed various peer-based strategies, including support groups, women’s social groups, counselling, and skill-based workshops (*see* Fig 2), focusing on holistic wellness, faith-based practices, nutrition, and mental health. SAMW suggested coaching to build social-emotional skills and regular group activities like swimming, walking, and hiking. Social groups and recreational activities were recommended to support mental health and alleviate loneliness, especially for the elderly. One female RL suggested targeted health initiatives for younger girls to develop positive lifestyle habits early in life.

In addition to peer interventions, some respondents recommended health education by community-based health professionals and RLs due to existing trust, respect, and shared values. These influential figures could be trained to deliver workshops on mental health, nutrition, women’s health, and noncommunicable diseases.

*“…Offer training, incentives and education to the people already out there, working, well-known, respected…empowering them, allowing them to give back to their community...”* ***(Participant 9, 18-29, Female RL)***

Interviewees also specified favourable peer and intervention characteristics that could impact the acceptability, reach, and effectiveness of interventions:

#### 3.4.1. Peer characteristics.

Favourable peer attributes specified by SAMW included role modelling, effective communication, competence, trustworthiness, and friendliness, with an emphasis on proper training. A non-judgmental and empathetic approach was seen as crucial for building rapport.

*“There are women out there who, like me, need that little push…or a friendly ear…to get them to come around and participate…”* ***(Participant 7, 60-65, Supervisor)***

RLs stressed the importance of passion, commitment, training, innovation, and confidence. Peer leaders must be accommodating and patient, as they may have to face challenging situations or undue criticism while doing community work.

*“They need to be approachable, amenable…patient and personable. When you do community work, you get a lot of stick…”* ***(Participant 15, Male RL)***

#### 3.4.2. Intervention characteristics.

SAMW emphasised the need for consistent and ongoing health programs, noting that previous initiatives were often discontinued. RLs also underscored the importance of long-term interventions for achieving sustainable impact. Timely promotion of events was seen as essential by women, allowing them time to organise their affairs, such as childcare planning. For support groups, women preferred a comfortable setting that fostered trust and confidentiality, particularly for discussing sensitive matters.

*“A support group where…you feel safe that whatever you talk to them about will stay within those four walls…”* ***(Participant 7, 60-65, Supervisor)***

A female RL shared her successful experience of organising women’s courses in an IRS, which incorporated faith-based health teachings and embraced an interactive approach.

*“We made a comfortable setting…provided refreshments… They had time to speak to people during the breaks…It was engaging, with life stories....”* ***(Participant 10, 40-49, Female RL)***

RLs suggested customising outreach strategies to engage women from diverse backgrounds and leveraging IRS social media channels to run digital health awareness campaigns. While social media is effective for reaching younger women, older women and busy mothers may require alternative outreach methods. Suggestions to boost participation included door-to-door outreach, encouraging family and friends, and promoting events within IRS.

*“It’s a case of ‘not one thing fits all’… it’s about taking information to people, especially people who aren’t engaging with your religious centres...”* ***(Participant 9, 18-29, Female RL)***

#### 3.4.3. Proposed logic model for FBIs.

**Fig 3:**
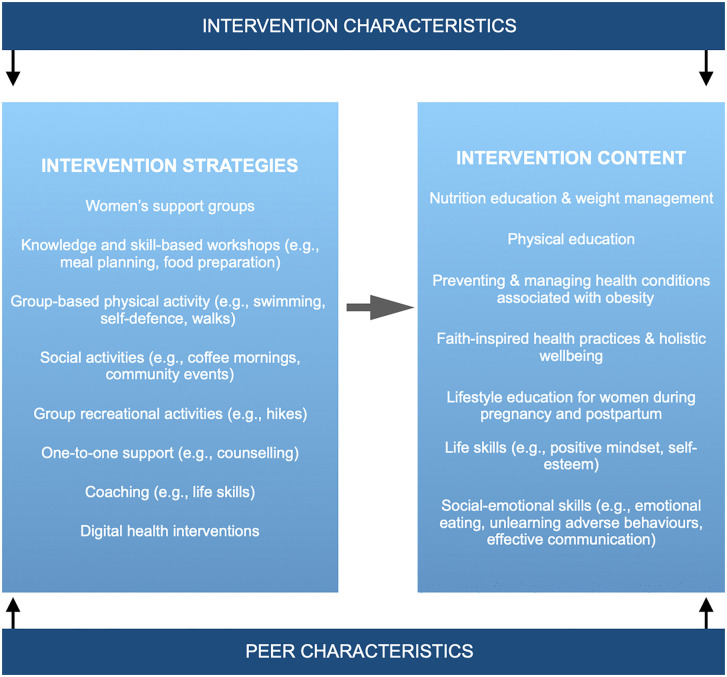
A simplified logic model for faith-based interventions in Islamic religious settings. The figure represents inputs, outputs, outcome, and desired impact. Contextual factors shape the intervention and may ultimately be impacted by the intervention (represented by two-way arrows).

A logic model [45] (*see* Fig 3), derived from the study’s thematic analysis, illustrates how the findings can guide the development of faith-based peer interventions in IRS. The model is grounded in the notion that these interventions are acceptable to SAMW, leveraging peers as credible sources of influence and social support, complemented by health education from professionals and RLs.

The model outlines the necessary inputs and activities required to achieve desired outcomes, such as improved health awareness, self-efficacy, social support, and healthier lifestyles, ultimately fostering community empowerment, shifting social norms, and reducing obesity-related inequalities. Contextual factors, including religious beliefs, social norms, entrenched inequalities, socioeconomic factors, and local policies influence and shape the intervention, and are in turn impacted by the intervention. The implications of this logic model are discussed in the following section.

## 4. Discussion

This study contributes to the sparse literature on FBIs for Muslim women. To our knowledge, it is the first study in England to examine stakeholders’ perspectives on obesity-related FBIs for women, as previous studies have largely addressed childhood [48] or adult obesity [20]. The findings support incorporating faith-based elements into obesity prevention programs, corroborating the evidence [20,48]. Religious beliefs emerged as a positive influence on participants’ lifestyles, while cultural factors shaped their dietary practices and attitudes towards health promotion. Understanding the social milieu and lived experiences of SAMW is necessary for designing effective FBIs. Peer-led interventions could be supplemented with health education from professionals and faith-informed teachings by RLs, as observed in prior interventions on cancer screening [28,29] and smoke-free homes [30]. The data underscore the need for accessible, consistent, and well-planned interventions, attuned to community values.

The findings indicate that the SA Muslim community experiences marginalisation due to inadequate funding and limited community engagement, with many community spaces being poorly maintained. SAMW are particularly vulnerable to health inequalities shaped by the intersection of religion, culture, gender, and ethnicity [49,50]. They may encounter negative stereotypes and discrimination based on their racial and religious identities [49,50]. Modestly dressed and visibly identifiable female interviewees described instances of feeling anxious and uncomfortable in community spaces, which restricted their participation in mainstream health and recreational facilities. They expressed the need for religiously sensitive, inclusive spaces for health activities, echoing findings in existing literature [51,52].

FBIs within IRS can provide a culturally and religiously congruent alternative to mainstream health interventions. However, factors such as leadership, funding, and women’s inclusion are key barriers to implementation. Cultural views on gender roles have traditionally hindered women’s involvement in IRS, but younger women are increasingly assuming active roles, likely influenced by higher education and evolving cultural norms. RLs also expressed keenness to support women’s inclusion, reflecting a generational shift in attitudes. While health promotion has been largely overlooked in IRS, which are often limited to worship and religious education, these settings have historically served as community hubs [53]. Transforming IRS into inclusive, holistic spaces is central to delivering FBIs. The varied satisfaction of SAMW with their local IRS hinted at the differences in cultural attitudes across settings and underscore the need to adapt interventions to each IRS.

Tailored health interventions can engage minority groups [18,54] and peer-to-peer approaches can minimise power differentials and enhance intervention acceptability [17,34] Peers can serve as educators, leaders, mentors, advocates, or support and outreach workers [34], fostering women’s self-efficacy and social support [17,21,28,29]. Such interventions can also strengthen SAMW’s engagement with health services by building trust, confidence, and health awareness [28,29,55]. Incorporating peer interventions into multi-level strategies is essential to address broader health determinants, such as organisational culture, social norms, unhealthy dietary practices, and food policies. A notable example is the REACH FAR (Racial and Ethnic Approaches to Community Health for Asian Americans) Project to control hypertension among Asian Americans [56]. Intervention components included healthy food policies for communal meals in religious settings to stimulate organisational change, and coaching to encourage individual behaviour change. Its success was attributed to linkages between RLs and community organisations, effective social marketing, training strategies, and the alignment with community values. Similar approaches could be adapted for Walsall-based IRS.

The study findings can guide the development of culturally sensitive health initiatives designed collaboratively with faith communities, public health agencies, and local groups. Intervention effectiveness and reach can be augmented through strong leadership, strategic planning, and structured volunteer training networks. Supporting peer educators through incentives, skill-building opportunities, and rewards, as well as providing training toolkits in native languages, can make interventions more scalable across diverse IRS. A model example is Birmingham City Council’s “Healthy Settings Toolkit”, designed for health promotion across six faith communities’ religious settings [57].

Community partnerships and cross-sector collaboration can facilitate resource sharing, pooling of funding and exchange of ideas. Co-designing interventions with communities promotes trust and local representation [17,58]. For example, a feasibility study on a culturally adapted African diet-based intervention found that co-developed healthy eating resources were practical, well-received, and potentially effective [59]. The Bradford childhood obesity trailblazer programme [60] (COTP) tackles childhood obesity among SAs through partnerships with IRS, organised training, and co-produced culturally sensitive toolkits to encourage healthy practices in IRS [61].The co-designed JU:MP programme, which encourages physical activity among SA girls while revitalising community spaces, exemplifies how cross-sector collaborations can thrive [62]. The need for a whole-system approach that accounts for the intersectionality between religion, culture, gender, ethnicity, and deprivation cannot be understated [58,63].

### 4.1. Strengths and weaknesses

This study’s strength lies in its diverse sample by incorporating perspectives from both RLs and SAMW. The recruitment of an equal number of male and female RLs helped minimise gender bias. The primary researcher’s shared religious, linguistic, and ethnic background with participants facilitated rapport, minimised power imbalances, and supported access to IRS, enabling a deeper understanding of participants’ perspectives and social contexts [43]. Although data analysis was conducted by a single coder, transparency was maintained during codebook development [64].

Nonetheless, the study faced limitations due to time and resource constraints. Despite extensive distribution of recruitment flyers, response was low – a common challenge in research involving ethnic minorities [65,66]. Low participation may have resulted from limited awareness of research relevance, disinterest, or competing demands, potentially introducing nonresponse bias. Consequently, the sample may have skewed towards women with greater religiosity, health consciousness and stronger views. Snowball sampling and social networks proved more effective for recruitment. Furthermore, the small sample size constrained the attainment of data saturation and may not have captured the full breadth of experiences within the target population; nevertheless, substantial repetition of core themes was observed across participants. The sample was also limited to English-speaking participants, thereby excluding non-English speakers and those with limited literacy, who may have different worldviews. Given that language proficiency often correlates with socio-economic status, education level, and health literacy [65,66], this may have influenced the findings.

### 4.2. Future research

Future research could aim for a more representative sample of Muslim women across different age groups to examine how age influences intervention needs, acceptability, and engagement. Including participants from diverse linguistic and ethnic backgrounds, particularly non-SA Muslim populations, would help determine whether the identified themes were consistent across cultures. This approach could also distinguish cultural factors from religious ones, given the heterogeneity within Muslim communities. Cultural influences could be further investigated, exploring the impact of societal norms and peer pressure on women’s self-esteem, body image, dietary habits, and motivation.

Additionally, perspectives from additional stakeholders such as local councils and public health bodies could provide insights into the challenges and opportunities for intersectoral collaboration. Given the study’s limited scope, additional research is warranted to translate identified barriers into systematic, actionable solutions that can be integrated into interventions. Exploring other community-centred approaches, particularly asset-based methods, to map community assets could support the development of holistic, multi-level interventions [17].

### 4.3. Conclusion

This study examined the potential of faith-based peer interventions to reduce obesity-related disparities among SAMW. While this approach appears viable, effective interventions should integrate faith teachings with scientific knowledge and be facilitated by trained individuals. Incorporating faith-based teachings can promote health awareness and challenge unhealthy cultural practices. With strong leadership, strategic planning, sustainable funding, and improved women’s facilities, IRS can evolve into holistic community hubs. Co-designing interventions with the community can ensure cultural and religious sensitivity, while embedding peer interventions within a whole-system approach is crucial for addressing structural inequalities.

## Supporting information

S1 File Interview guide. Condensed topic guide for semi-structured interviews.
(PDF)

S2 File COREQ Checklist. (PDF)

S1 Fig Coding Tree. Coding tree incorporating themes from both participant groups.
(PDF)

S1 Table Illustrative quotes. Illustrative quotes representing the coding tree.
(PDF)
