# Supplementary material for: Barriers and facilitators to developing faith-based peer interventions in Islamic religious settings for obesity prevention in women: A qualitative exploratory study
Source: PLoS One. 2026 Jan 5;21(1):e0340087. doi: 10.1371/journal.pone.0340087 (PMC12768345; doi:10.1371/journal.pone.0340087)
Supplement: S1 File — Condensed topic guide for semi-structured interviews. (PDF) [file pone.0340087.s001.pdf]

Supplementary File 1: CONDENSED TOPIC GUIDE FOR SEMI-STRUCTURED INTERVIEWS

Note: ***Female religious leaders*** *were interviewed about all these themes by combining both topic guides.*

	SOUTH ASIAN MUSLIM WOMEN	***MALE RELIGIOUS LEADERS***
INTRODUCTION:	➢ Introduction, rapport building, explain the interview structure, discuss confidentiality and take consent.	
WARM-UP:	➢ Understand participant's background, routine, key roles and responsibilities.	
CORE DISCUSSION: *Understand lifestyle behaviours and their determinants*	➢ *How do you feel about your health? What are the activities you like to do for your wellbeing?*<br>➢ *When you are deciding which foods to buy, what is most important to you?*<br>➢ *How active are you in your daily routine? Can you describe the ways in which you try to stay active?*<br>➢ *What are some of the challenges you usually encounter in trying to maintain a healthy lifestyle?*<br>➢ *How do you like to get information or advice regarding your health? What are the sources that you trust and follow?*	➢ **Not applicable**

	➢ *How do you feel about the available community resources to support your health?*	
Understanding the community context and the role of religious settings	➢ **Not applicable**	➢ *Can you tell me about the activities commonly organised in this religious setting?*<br><br>➢ *What are your thoughts about using religious settings for health-related activities?*<br><br>➢ *How do you feel regarding the participation of women in such activities?*<br><br>➢ *It would be helpful to hear about any past initiatives in religious settings to promote health. What did you think of the overall experience?*<br><br>➢ *How do religious settings keep up to date with the health issues of the community?*<br><br>➢ *Are health messages included in mosque communications?*<br><br>➢ *What do you think of the available community resources for promoting health and wellbeing?*
Views on faith-based interventions	➢ *What activities are typically organised in religious settings for the community?*<br><br>➢ *How do you feel regarding the inclusion of women in these activities?*	➢ *What is the role of faith in how Muslims care for their health? Do you feel that incorporating faith-based elements in health activities has any additional value?*

	➢ *Does the mosque support health-related discussions? Are health messages included in mosque communications?*<br><br>➢ *How would you feel about participating in a health activity/program in a religious setting?*<br><br>➢ *Are you aware of any past or current health initiatives in local religious settings? What did you think of the overall experience?*<br><br>➢ *What role does your faith play in how you care for your health? What are your thoughts about incorporating faith teachings in health programs?*<br><br>➢ *Explain peer interventions and ask the following questions:*<br><br>*How would you feel about participating in peer-led activities in a religious setting? What factors could influence your decision to participate?*<br><br>*Can you think of any other ways to use faith/ religious settings for improving women's health in the community?*	➢ *Explain peer interventions and ask the following questions:*<br><br>*What are your thoughts regarding organising peer activities in religious settings to promote women's health? What factors are important to consider when planning such interventions?*<br><br>*Do you feel that religious settings have the capacity to organise such activities for women?*<br><br>*Are health and wellbeing projects usually discussed in administrative meetings? Does this religious setting have a vision/plan for promoting community health?*<br><br>*Has the management considered any practical ways to promote community wellbeing?*<br><br>*Does this religious setting have any partnerships with local groups and services to facilitate the organisation of such health projects? Do you have suggestions on how to improve these partnerships?*
WRAP UP:	➢ Offer participants the opportunity to give comments or ask questions.<br>➢ Thank participants. Reiterate confidentiality. Close the discussion.	
