# Supplementary figures and images for: Barriers and facilitators to developing faith-based peer interventions in Islamic religious settings for obesity prevention in women: A qualitative exploratory study
Source: PLoS One. 2026 Jan 5;21(1):e0340087. doi: 10.1371/journal.pone.0340087 (PMC12768345; doi:10.1371/journal.pone.0340087)

# Supplementary Fig 1: CODING TREE INCORPORATING THEMES FROM BOTH PARTICIPANT GROUPS

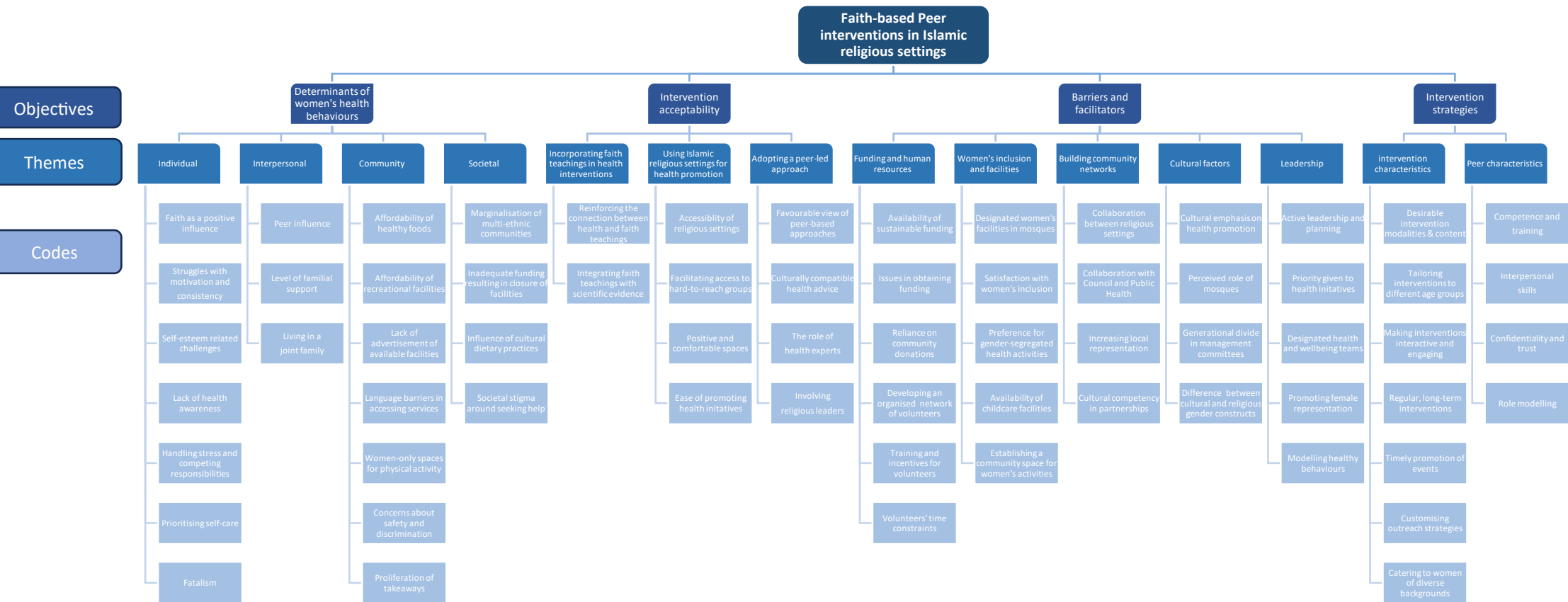

Supplement: S1 Fig — Coding tree incorporating themes from both participant groups. (PDF) [file pone.0340087.s003.pdf]
