# Supplementary material for: Barriers and facilitators to developing faith-based peer interventions in Islamic religious settings for obesity prevention in women: A qualitative exploratory study
Source: PLoS One. 2026 Jan 5;21(1):e0340087. doi: 10.1371/journal.pone.0340087 (PMC12768345; doi:10.1371/journal.pone.0340087)
Supplement: S1 Table — Illustrative quotes representing the coding tree. (PDF) [file pone.0340087.s004.pdf]

Supplementary Table 1: ILLUSTRATIVE QUOTES REPRESENTING THE CODING TREE

**Determinants of women's health behaviours**			
**Themes**	**Codes**	**SA Muslim women**	**Religious leaders**
**Individual factors**	Faith as a positive influence	*"I'm motivated by my religion to eat healthy…Prophet [Muhammad]…would encourage exercises…sports, horse riding, archery… [Our religion] talks about giving yourself time…"*<br><br>*"Islam teaches us that we shouldn't overeat, overindulge, shouldn't eat more than your fill…I try to implement that. "*<br><br>*"We believe that our body and our health is a trust from God…It motivates me to look after my body whether it's my physical health or my mental health, or even just…remaining clean and pure. …We have to nourish it with things that benefit us."*	*"Islam certainly says that we have to look after our health as best as we can because it is a gift from God."*
	Struggles with motivation and consistency	*"When I try a new regime…to become healthier…I give up too easily if it's not showing instant results…Let's say just a bad day happened or it's busy… it's back to square one again."*<br><br>*"If I eat processed foods, that's only because it's the quickest, easiest thing to eat right now..."*	

		"If you want to to live a better quality of life you have to push yourself. And I think I don't push myself [enough]…"	
	Self-esteem related challenges	*"When it comes to fitness and moving your body, it's just…people seeing me in that setting, I'd probably feel a bit less confident, and think twice."*<br><br>*"I'm not the kind of person that likes going to the gym and working out in front of other people."*	
	Lack of health awareness	*" I think it's a lack of education on nutrition and… physical health. People don't even know that they're not eating optimally."*<br><br>*"When a woman gets pregnant, if she's coming to you, tell her how important it is to eat healthy in pregnancy. They think that they're [only] gaining weight because of the baby."*	
	Handling stress and competing responsibilities	*"Unfortunately, my issue is the inability to handle stress properly."*<br><br>*"With stress, the urge of food sometimes can be very powerful… that in the end you give in to it."*	*"When they get overwhelmed, I think that's when they lose their whole… like…looking after themselves."*
	Prioritising self-care	*"If you don't have time for yourself…[When] you're hungry, whatever is available, you grab it and you eat it… First you need to love yourself. You need to give yourself time. You need to give yourself priority which is lacking in our community."*	*"I know now women are looking after themselves in the younger generation, but I think in the older generation there is that concept of just catering to everyone else and not looking after themselves."*

	Fatalism		*"What we [the community] are not good at is working on prevention, and I think it's partly to do with our belief that regardless of what we do, it is going to happen to us [our destiny]."*
**Interpersonal factors**	Peer influence	*"I think me putting on weight has been because of my environment…Everybody just making you feel that: 'Hey, you're not fat…' For me, my reality check was seeing a photo of myself and I was like, 'What, is that me?'"*<br><br>*"When I'm doing a gathering, I am forced to put more oil, more salt… Once I came to know that someone says she is not willing to put some extra money by putting some extra oil in the food."*<br><br>*"We have a group online where we all motivate each other with a group of sisters…about our health, our work, our nutrition, and everything…"*	
	Level of familial support	*"My husband loves to have oil in his food, so I have to cook something separately for myself, which is quite challenging."*<br><br>*"At the moment when I do exercise, it's a punishment…I feel that I'm the only one who's doing it."*<br><br>*"I cook for my family, so I'm in charge…It's very easy for me to change the kind of cooking, and no one minds..."*	

	Living in a joint family	*"If I was in my own space, I'd be more disciplined because I've got less distractions and influences around me, less people pleasing…"*<br><br>*"When meal planning, because I'm in a joint family system I don't really worry about dinners because that's covered. But, it's not always very healthy."*	
**Community factors**	Affordability of healthy foods	*"I know personally in the community so many people [who] struggle to buy ingredients which are good for health. Even if you go to food banks, they have tinned foods..."*<br><br>*"It's a myth that when people say it's expensive…because you can buy single fruit from any supermarket… for 20p, 30p…There's always an option, but only if you want that option…"*	*"With the cost-of-living crisis ...there are families who are finding it difficult to survive. And in terms of providing healthy options, it is very difficult and not affordable."*
	Affordability of recreational facilities		*"Walsall is a very underprivileged area…There are gyms, sports centres, leisure centres, but unfortunately a lot of them are quite costly and I think for a large proportion of our community they're just not accessible and affordable."*
	Lack of advertisement of available facilities	*"I feel that there's a lack of easily available information if there are any facilities here."*<br><br>*"I would say door to door advertising would really help…or posters of these things at local mosques."*<br><br>*"I know there might be something running in the community centre, but I don't know where*	*"I don't think there's enough information and enough access for people to these facilities."*

		to access all that information besides physically going myself, which is not me."	
	Language barriers in accessing services	*"If I were to look at the women in my family, a lot of them would not feel comfortable engaging in these sorts of services due to the fear that they wouldn't fit in, that they wouldn't understand…"*	*"A lot of our community still speaks Urdu, Mirpuri, other languages that aren't as accessible within the NHS, you know, require translator, which puts up its own barriers. I think that doesn't help with engagement."*
	Women-only spaces for physical activity	*"As a female Muslim who observes hijab…Ideally for me, the class would have to be held my female and also a space that allows me to cover and feel comfortable…"*<br><br>*"I've always enjoyed the ladies-only gyms and things like that."*	
	Concerns about safety and discrimination	*"…As a female who doesn't drive…we don't really live in a safe area…I'd be fearful of something happening…"*<br><br>*"When I went to gym [in religious attire]…people were [staring at me] as if I am here to show some circus…I never went back again."*	*"Our women, especially if they wear hijab (head covering), feel vulnerable, they don't feel safe... We would encourage women to go in groups."*
	Proliferation of takeaways	*"We do have a lot of takeaways here…Once a week or sometimes even twice, I eat out at lunch time. And I know it's not the healthiest."*	*"If you look at the number of takeaways that have cropped up… where largely…we have a Muslim community living…so of course that is adding to all the problems."*
**Societal factors**	Marginalisation of multi-ethnic communities		*"People from these communities have faced a lot of issues…whether that be isolation, discrimination…alienation, poverty…Those are just some of the issues that our ethnic minority communities have faced. So, there's almost a natural scepticism."*

			"A lack of engagement with the community and having one blanket approach for all…"
	Inadequate funding resulting in closure of facilities	*"If you look at even most community halls, they are closing them down. There are not enough community halls…"*	*"Walsall Council has faced a lot of cuts…If we went back ten years ago, there was a lot more out there for the community. We had youth clubs…more community centres…baby and mother centres…"*<br><br>*"[Sure Start] was a vital service…You were educating mothers about the early years, which led to better habits, lifestyles, choices and routines within the family unit…"*
	Influence of cultural dietary practices	*"Health is not first, it's not prioritised. And so, it will be a culture of eating. Our food is very heavy, usually fried, and we have these things multiple times a day… It's always around food, then you don't even have time to move."*<br><br>*"Culture is the key factor. You're used to something already, so you don't question it. You're used to a certain lifestyle, and you find nothing wrong with it."*<br><br>*"When we go to weddings or parties... all have unhealthy foods. There's nothing [healthy] there for you to eat."*	*"We often use the same practices, the same ingredients to make our foods as we did back in [our home country]. Unfortunately, in this weather, looking at our lifestyle, looking at what we do, [the food we eat] isn't processed in the same way."*
	Societal stigma around seeking help		*"As a community, we're not accessing some of the…help from the Council and other charitable organisations…because of either lack of education. Or because…they don't want to be seen begging…It's part of our traditional heritage. Sometimes we suffer in silence."*

Intervention acceptability			
**Themes**	**Codes**	**SA Muslim women**	**Religious leaders**
**Incorporating faith teachings in health interventions**	Reinforcing the connection between health and faith teachings	*"If you can't be the best version of yourself, you can't follow your religion properly."*<br><br>*"People always pick and choose what they want…It's the same with health…But I think if they go back to the basics [of faith]…probably 50% of their illnesses would go [away]."*	*"A strong believer is better than a weak believer. Our looking after our health is an important part of our Imaan [faith] as our spiritual well-being and physical well-being go together."*<br><br>*"Faith plays an important role in health. But it is a balance between the two. If you can explain the balance between the two then…people will take it on board more than talking about health solely on its own."*<br><br>*"Most, if not all, faiths promote health and… looking after yourself…and I think…it's important to get that message across and remind people that this is what their faith says."*
	Integrating faith teachings with scientific evidence	*"We could have dieticians, nutritionists, they would come and talk about food but then they would bring in all the things that they know from Sunnah [the practices of our Prophet]."*	
**Using Islamic religious settings for health promotion**	Accessibility of religious settings	*"Because everyone is linked to the mosque, and…everyone has access to it…It would just make it easier for people to get more out of their religious spaces."*	
	Facilitating access to hard-to-reach groups		*"You will address… you'll engage with people you wouldn't otherwise engage with."*

			"If you're targeting Muslim women and those who would not maybe make use of other facilities, then having [activities] in these religious settings is probably making it more accessible and comfortable for them."
	Positive and comfortable spaces	*"In a religious space…we're allowed to feel as if we're all the same…I would definitely…feel a lot more comfortable."*<br><br>*"Obviously a mosque is a positive space for us Muslims and if the mosque is promoting such a thing, it would draw more people to go and take part."*	*"They feel a lot more comfortable when we're running these events and there's faces that they know that will be present…"*
	Ease of promoting health initiatives		*"…Majority of the people are more linked with mosques than other organisations…If you've got 500 people coming to mosque daily, to promote all activities…is much easier…".*
**Adopting a peer-led approach**	Favourable view of peer-based approaches	*"I think it's good if it's coming from the same background because then they have an understanding of South Asian culture, lifestyle, diet, health…"*<br><br>*"Even within our Muslim community, we have different cultures….So sometimes people are more attracted towards those who are from your own culture."*	*"If you were to have somebody from within your own community…who has shared your experiences…. who speaks your language…I think that becomes a very natural, trusting experience…"*
	Culturally compatible health advice	*"The [non-Asian] coach does give a nutrition plan, but I find that very hard to stick to… because I'm used to our Asian foods…I didn't enjoy the taste…"*<br><br>*"If there's someone from the community… when they share healthy ideas, then it's easier*	

		to incorporate that because we already eat the same food."	
	The role of health experts	*"Where somebody would be a stranger and this person would be known to the community already, and especially if they were already an expert in that area."*<br><br>*"They [peer educators] themselves need their professional back up…to say, 'Look, I'm here because I got support from somebody else and I'm here to support you as a community member.'"*	*"I think it has to come from within the faith community. So, from doctors, healthcare professionals and educators within that community helping their own people."*<br><br>*"If we have doctors and nurses that are from this community, it's about approaching them and giving them the skills and tools to do more health promotion within their community."*
	Involving religious leaders		*"Empowering religious leaders, people that are already working with the community…training them, educating them, allowing them to give back to their community and…do those health promotions."*

Barriers and facilitators to faith-based peer interventions in Islamic religious settings			
**Themes**	**Codes**	**SA Muslim women**	**Religious leaders**
**Funding and human resources**	Availability of sustainable funding		*"Running promotional and health events is not cheap…You need…space, appropriate resources… We are not getting (government) funding."*<br><br>*"[Funds] can be an issue because obviously we are not getting funding for running these religious centres from governments."*
	Issues in obtaining funding		*"As a Muslim organisation, we have to be careful about where the funding comes from…A lot of the funding available these days is lottery funded. As a mosque, we cannot take the lottery funding."*<br><br>*"…Those mosques that are not open to the whole community, they find it difficult to access public funds."*<br><br>*"There is funding available from Muslim organisations and… it's lack of knowledge that we can't access that."*
	Reliance on community donations		*"The way mosques are run financially, they are healthier than other organisations because you have…a membership system. You have people affiliated with the mosque... So, it's much easier financially to run these sorts of projects."*

			"Sometimes financially if you need help, you can make announcements…and people are willing if they know that there's a good project."
	Developing an organised network of volunteers	*"We don't have any network so that we could better our own communities… It's not that there's a lack of talent within the community...It's just so scattered that…it's not being utilised…I think it's the job of religious leaders to look for these people..."*	
	Training and incentives for volunteers	*"If I'm [volunteering]…my family will say, 'Where are you spending your time?'…At the end of the day, if I bring some money, they won't say anything to me."*	*"We need to encourage some kind of payment for the volunteers. Because we want the volunteers to be on time…that they are encouraged to complete [their tasks]. One of the successes of the COVID-19 champions scheme that we ran with the Mosque was that we actually paid the volunteers."*
	Volunteers' time constraints		*"Our downside is we're all volunteers. [We] can only give the volunteer level of commitment."*<br><br>*"If you look at the structure of the mosque, it's all volunteers…What they haven't got the time for is really to sit down and say…, what are our priorities."*<br><br>*"I think we've had massive team changes, lots of people have gone away…Unfortunately where you have an organisation that's dependent on volunteering then we face a major hit."*
**Women's inclusion and facilities**	Designated women's facilities in mosques		*"One step is to actually provide a dedicated space for them, a safe space…"*<br><br>*"We are encouraging mosques to be open to everyone because, as you know, some of our mosques are not open to women."*

	Satisfaction with women's activities	*"They're not included most of the time. And what is available is very limited. So, either it's at a time where most people cannot attend, which is like weekdays in the morning. Or…there's so little variety."*<br><br>*"…It's such a loss for the community if women are not given equal access to the things that they could be benefiting from by being an active part of the mosque… Where are they supposed to go?"*<br><br>*"Mosques are the only places not getting any funding from government… So if you look at it that way, they are doing a really good job because no one is helping them."*	*"Women have more opportunities compared to a few years ago. But I wouldn't say it's a good amount of opportunities. I feel there should be more for women available in this area because so many women are connected to the mosque."*
	Preference for gender-segregated health activities		*"We tend to run them (events) separately because we find that women are more comfortable about opening up and questioning the professionals there..."*<br><br>*"Women will feel more comfortable if a woman is there and taking up these projects and giving awareness programmes."*
	Establishing a community space for women's activities	*"I do feel that… we would need a third space because majority of the religious spaces are very small…. And if you have a [third] space like that, then you'd be regular in having those [activities]…"*	*"A lot of religious centres…don't have community spaces. So, I think actually having the space, finding a room which is big enough to host an event…host a group of people."*
	Collaboration between religious settings		*"Maybe if projects weren't limited to certain mosques…They were…pushed in as many mosques as possible."*

**Building community networks**	Collaboration with Council and Public Health		*Having that link with the Council…that plays a very, very important role.”*<br><br>*“It needs to be a joint vision with the public health, with the local community and there needs to be recognised tangible outcomes.”*
	Increasing local representation		*”We need active organisations in the SA communities to work with public health and put pressure on the service providers…to be active in this area, much more than they currently are.”*<br><br>*“it's really getting the need that we have channelled in the right direction so that public health do actually make some of these conditions in the South Asian communities a priority.”*
	Cultural competency in partnerships		*“[Describing a partnership with SureStart] We were very comfortable with them even though we wore [face veils] …They were very friendly, accommodating…A non-judgemental space...”*
**Cultural factors**	Cultural emphasis on health promotion		*“Those who are in the management and leadership roles…[They] are reluctant to start these projects because they don't see much of a demand.”*<br><br>*“I don't think it's given enough emphasis at all within the Muslim community, within the Asian community…. And it's something that needs to be pushed.”*
	Perceived role of mosques		*“In the time of Prophet Muhammad …(Mosque) wasn't just a place of prayer and contemplation…It was the hub of the community. So why shouldn't our mosques become the hub of the community?”*

			"Where there's demand, then projects, programmes will take place… Probably people are just not interested or they don't really feel that mosque needs to plays a part in health or health awareness."<br><br>*"90% of the masjids are standing empty 70% of the day. So actually, if you've got the facility there, why can't we use it during the rest of the day?"*
	Generational divide in management committees		*"We have challenge from the elder generation who have a different view…about what the mosque should be…They are now coming around to our way of thinking..."*
	Difference between cultural and religious gender constructs	*"[In Islamic history], women were really active in the community. Culture is… they make it [seem] that women might need to stay at home, or they can't do anything. But…Islam is not like that."*	*"I think in older generations there's probably a massive reluctance to come forward because they don't feel that that's their place in society. But I think if you were to look at more younger generations who have very different cultural mindset, there's much more active efforts to make a place for themselves in community."*
**Leadership**	Active leadership and planning		*"It all depends on…[the] people who lead…The imams that have a vision, they're very active. So those [IRS] will probably have a lot of programmes…"*<br><br>*"Imams play a very critical role in this, not just leading the prayer or giving lectures, but also to the wider community."*
	Priority given to health initiatives		*"Our mosques are already so burdened by the number of (community) issues they have to deal with day in and day out that health goes very far down the list."*

	Designated health and wellbeing teams		*"Having a separate team that's built within the mosques that focuses on those projects, that maybe would help mosques to…give more to the community in regard to health."*<br><br>*"I think if we had maybe an active team that was responsible for making sure that …every quarter there was something running at the local mosques."*
	Promoting female representation		*"…I remember growing up, the resources and the inclusion of women was very poor…In SA communities, there's often a cultural mindset that men are…the ones at leadership [roles]…"*<br><br>*"Our make-up is almost 100% men. So, we've created a subgroup for women that works with the executive committee…We are very, very keen to ensure the representation of [women]."*<br><br>*"Certainly, we need to establish women groups in in each of the mosque that looks at the health inequalities amongst women."*<br><br>*"There are mosques that are very far behind when it comes to giving sisters equal opportunities as the men, in terms of running things or having access to certain things."*
	Religious leaders modelling healthy behaviours	*"…Most of the Imams…they're obese…They are definitely not leading by example…I'd like to see them not serve pakoras and samosas at every event."*	

**Intervention strategies**			
**Themes**	**Codes**	**SA Muslim women**	**Religious leaders**
**Intervention characteristics**	Desirable intervention modalities and content	*"Teaching people about how to manage their lives better and eating better and incorporating more fitness…even meal planning for people because some people just don't know how to incorporate something different into their life."*<br><br>*"Mentoring, coaching or mental health support or, you know, just somewhere for women to hang out."*<br><br>*"I would like to have something that is more, a group effort because it's easier to do when you have more people."*<br><br>*"I think if they have groups where they are encouraged to go have little walks or maybe exercise sessions together."*	*"A lot of the women want to become active and look after their health… They want something actually physical, so they want swimming.. exercise classes, maybe self-defence classes. They want a gym to use. That's what they're looking for. They're looking for an actual kind of provision of an activity rather than workshops and education."*<br><br>*"We need to offer alternatives. We need to offer ways in which women, for example, can cook. We need to offer more cooking classes…educate the community about where best to buy foods."*
	Tailoring interventions to different age groups	*"I feel like we need to start working on people at a younger age and give them access to these workshops because they need to understand the long term effects [of unhealthy behaviours]."*	*"You can't tell your average 50-year-old woman to go to a gym, that's just not something that would work for them. So, it's about offering alternatives…Will it be a support group that does weekly walks in the park, for example? Will it be something that's held within the Mosque?"*
	Making interventions interactive and engaging	*I sometimes think that with talks, some people, they're not really focused in it... Even just having morning coffee where everybody talks and sort of, bring out a conversation. That'd be*	*"We made a comfortable setting…provided refreshments… They had time to speak to people during the breaks…It was engaging, with life stories...."*

		a good way rather than someone just speaking to a group of people."	
	Regular, long-term interventions	*"Something that…would continue, something regular. Yeah, because sometimes you have… a one-day thing. Some event for woman, and then never happens again."*	*"We need it for a longer period. I would say a minimum of three to five years intervention and funding of peer-to-peer is needed."*
	Timely promotion of events		*"Advertising beforehand not leaving it last minute…People need time to organise things and…make sure that they have availability or they have childcare available."*
	Customising outreach strategies	*"Within my close community, I see that old ladies need a bit more encouragement to leave, I think because they've been inactive for a while now, so…bringing them out would be good."*	*"…Reaching out to these communities, acknowledging their discomfort, working with them to come up with solutions… What works for you? ...Whether that be sessions for religious communities separately…at their places of engagement…in their languages…"*<br><br>*"It's a case of 'not one thing fits all'… You have to really vary the way you're promoting your events to your different sections of the community…It's about taking information to people, especially people who aren't engaging with your religious centres..."*<br><br>*"Advertising and promoting and really making people understand the importance. I think it took a lot of active effort to reach out to all different ages within the community and get people to come along."*
	Catering to women of diverse backgrounds	*"What is available is very, very limited. So, either it's at a time where most people cannot attend, which is like weekdays in the morning. Or, there's so little variety."*	

		"I can't go to those circles because they're in the daytime when I'm at work..."<br><br>*"You need to cater to all different types of women. The women that work, the women who have small children, creche facilities…Different age group appropriate activities."*<br><br>*"Maybe through social media trying to gather people who would be interested…and doing a survey of the time that would suit most people."*	
**Peer characteristics**	Competence and training	*"I'd like them to have some passion… knowledge…I'd like to go to the right place for the right information."*	*"I think they need to be trained up to the highest level so they're able to provide the information that's needed."*<br><br>*"…Offer training, incentives and education to the people already out there, working, well-known, respected…empowering them, allowing them to give back to their community..."*
	Interpersonal skills	*"There are women out there who, like me, need that little push…or a friendly ear…to get them to come around and participate…"*<br><br>*"They would have to be non-judgemental, sympathetic, caring… because sometimes it's scary for people to talk to someone about what they're going through."*	*"They need to be approachable, amenable…patient and personable. When you do community work, you get a lot of stick…"*<br><br>*"It's just about reaching out to people... And you got to have such people…who have good community links, people who are able to speak and…talk to other people."*
	Confidentiality and trust	*"A support group where…you feel safe that whatever you talk to them about will stay within those four walls…"*	

	Role modelling	*"They have to lead by example. You can't be telling us about nutrition if your own life does not reflect that."*	
